# Animal Models of CMT2A: State-of-art and Therapeutic Implications

**DOI:** 10.1007/s12035-020-02081-3

**Published:** 2020-08-27

**Authors:** Roberta De Gioia, Gaia Citterio, Elena Abati, Monica Nizzardo, Nereo Bresolin, Giacomo Pietro Comi, Stefania Corti, Federica Rizzo

**Affiliations:** 1 Neurology Unit, IRCCS Foundation Ca’ Granda Ospedale Maggiore Policlinico, Via Francesco Sforza 35, 20122 Milan, Italy; 2 Dino Ferrari Centre, Neuroscience Section, Department of Pathophysiology and Transplantation (DEPT), University of Milan, Milan, Italy

**Keywords:** CMT2A, MFN2, Animal model, Strengths and weaknesses

## Abstract

Charcot–Marie–Tooth disease type 2A (CMT2A), arising from mitofusin 2 (*MFN2*) gene mutations, is the most common inherited axonal neuropathy affecting motor and sensory neurons. The cellular and molecular mechanisms by which *MFN2* mutations determine neuronal degeneration are largely unclear. No effective treatment exists for CMT2A, which has a high degree of genetic/phenotypic heterogeneity. The identification of mutations in *MFN2* has allowed the generation of diverse transgenic animal models, but to date, their ability to recapitulate the CMT2A phenotype is limited, precluding elucidation of its pathogenesis and discovery of therapeutic strategies. This review will critically present recent progress in in vivo CMT2A disease modeling, discoveries, drawbacks and limitations, current challenges, and key reflections to advance the field towards developing effective therapies for these patients.

## Introduction

Charcot–Marie–Tooth type 2A (CMT2A; OMIM 609260) is a dominant inherited sensory motor neuropathy that affects peripheral nerve axons and is characterized by a heterogeneous phenotype including not only neuropathy-related features but also systemic impairment of the central nervous system (CNS) [1, 2]. Motor symptoms not only are predominant in the distal lower limbs, but they may also involve distal upper limbs in half of the diagnosed cases [3], leading to progressive muscle weakness, foot deformities (*pes cavus*), gait disturbances, and areflexia. Other typical features are sensory impairment, mainly affecting vibratory sensation and proprioception and neuropathic pain [4]. CMT2A may also lead to sensory neural impairment, such as hearing loss [5, 6] and other clinical features, such as hoarse voice, vocal cord paresis, and signs of respiratory insufficiency [2].

CMT2A is caused by missense mutations in the *mitofusin 2* (*MFN2*) gene, which encodes a GTPase dynamin-like protein localizing to the outer mitochondrial membrane that is mainly involved in the regulation of mitochondria-related processes, such as mitochondrial fusion, mitochondrial transport along axons, and mitophagy [7–11]. Furthermore, MFN2 participates in mitochondrial metabolism and intracellular signaling [12].

Although few recessive forms have been described [13, 14], CMT2A is generally associated with dominant mutations distributed along the *MFN2* sequence [7, 15–19]. Dominant mutations may lead either to a gain or to a loss of function according to the position of the mutation within MFN2 domains [20]. In particular, *MFN2* mutations seem to induce disease with a “dominant-negative” mechanism, where the expression of the wild-type *MFN2* allele is negatively regulated by the mutant protein. To date, no *MFN2* mutations have been associated with haploinsufficiency. The cellular and molecular mechanisms by which *MFN2* mutations determine neuronal degeneration are not fully understood. However, recent progress revealed that multiple mechanisms contribute to pathogenic *MFN2*–related axonal degeneration including alteration of mitochondria transport and localization [10, 21–25] and mitochondrial–endoplasmic reticulum crosstalk impairment [25, 26].

Considering the high degree of genetic and phenotypic heterogeneity of CMT2A and the involvement of motor and sensory components in disease pathogenesis, the generation of reliable models is very tricky but essential for studying disease pathogenesis and finding resolute therapeutic approaches, which are currently unavailable. Unfortunately, until now, few in vitro and in vivo CMT2A models have been developed.

Regarding in vitro models, patient-derived motor neurons (MNs) [10, 27] are a more effective disease model than previous ones based on non-human primary cell lines (e.g., mouse embryonic fibroblasts (MEFs) and mouse dorsal root ganglia (DRGs)) [16] and on cell types that are not disease-relevant (e.g., fibroblasts) [23, 24]. However, sensory neurons (SNs) derived from CMT2A patients have not yet been generated, representing a restriction to modeling this disease characterized by the involvement of both motor and sensory components [16, 23, 24].

Regarding in vivo models, transgenic models of Drosophila and zebrafish have been helpful for demonstrating the function of Mfn2 in mitochondrial dynamics and its role in progressive loss of motor function [28–30].

However, mammalian models could represent a more relevant tool for human disease, although there are physiological, genetic, and technical limitations to the use of rodents to model hereditary neuropathy. The possibility of obtaining a good and relevant disease model depends on the mouse strain and its genetic background, which cannot interfere with the phenotype, and on the promoter of the transgene, which should allow moderate expression of the target protein to avoid artifacts linked to overexpression. Furthermore, the development of such models and their phenotypic assessment are time-consuming, and the development of the expected phenotype can never be guaranteed.

This review will provide an update on the in vivo murine disease models (Table 1) developed to date, critically discussing how they could be improved and their involvement in the development of novel therapeutic strategies.

**Table 1:** Comparison between available CMT2A mouse models

	*HB9 Mfn2*^*T105M*^ (Detmer 2008)		Eno *MFN2* ^R94Q^ (Cartoni 2010)		Nestin-cre *MFN2*^T105M^ (Bannerman 2016)		Thy1.2 *MFN2*^R94Q^ (Zhou 2019)
*MFN2* mutation	T105M		R94Q		T105M		R94Q
MFN2 transgene promoter	Hb9		Eno		Rosa-STOP-MFN2T105M/CAG-CreERT2	Nestin-cre	Thy1.2
Genotype	Homozygous	Heterozygous	Homozygous (MitoCharc2)	Heterozygous (MitoCharc1)	Homozygous	Heterozygous	Y-linked
Phenotype onset	Severe congenital	Mild congenital	Mild late	Mild late	6 W post-tamoxifen induction	Mild late	Early
Motor resistance (Rotarod test)	Hindlimb muscles weakness but no alteration	No alteration	Frequent fall off	Frequent fall off	Not detectable	Not significant	Frequent fall off
Grip strength	/	/	/	/	/	/	Frequent fall off
Gait (Noldus Catwalk)	Defect in dorsi-flexion but no gaiting alteration	No alteration	Abnormal print length	No alteration	/	Abnormal print length	Progressive gaiting worsening
Axon (number, size, g-ratio)	40% fewer axons in motor roots (L4 and L5)	No alteration	+ 55% of < 3.5 μm axons	+ 40% of < 3.5 μm axons; Aδ fibers altered in the sciatic nerves	/	No alteration	Degeneration in tibialis muscle
Muscle fiber	Smaller anterior hindlimb muscles	No alteration	No alteration	No alteration	/	Smaller tibialis and soleus muscles	/
Mitochondria (number, aggregates, axonal transport)	Highly aggregation and clusters	Highly aggregation and clusters	N° mitochondria + 28% in < 3.5 μm axons	N° mitochondria + 34% in < 3.5 μm axons	Reduced number in tibialis axons	No alteration	Clusters and morphology abnormalities. No mitophagy
Sensitive phenotype	/	/	/	/	/	/	/

## Mouse Models of CMT2A: Strengths and Weaknesses Affecting Clinical Application

### *HB9 Mfn2*^*T105M*^ Transgenic Model

In 2008, Detmer and his group generated the first CMT2A transgenic mouse model expressing the pathogenic murine *Mfn2*^T105M^ allele under the homeobox HB9 promoter in peripheral MNs [31]. The *Mfn2*^T105M^ mutation, located in the middle of the GTPase G1 motif of MFN2, was shown to cause early-onset lower limb muscular atrophy as well as scoliosis and ataxia in three unrelated CMT2A families [7, 32]. This mutated protein localizes properly to mitochondria but is defective in mitochondrial fusion and induces mitochondrial aggregation when overexpressed [18, 21]. Given the data in cultured cells [18, 21], MN expression of these mutated *Mfn2* alleles in mice might be expected to lead to a reliable animal model to recreate CMT2A clinical motor defects. Heterozygous and homozygous *Mfn2*^T105M^ transgenic mice showed different transgene expression levels, and heterozygous animals developed a much milder phenotype, suggesting that the phenotype depended primarily on transgene expression levels. Regarding motor phenotype, this model recapitulated some of the motor defects observed in CMT2A patients: homozygous transgene mice showed severely deformed and remarkably shorter tails, as well as gait defects evident from birth. The majority (86%, *n = 85*) of these animals were unable to perform dorsiflexion of the ankle and dragged their hindlimbs while walking. Similarly, paresis of the tibialis anterior is the most consistent clinical sign in CMT2A patients, determining a foot drop–related gait impairment [33]. In addition, homozygous animals often present clenched hind paws due to an apparent inability to spread the toes. Despite these defects, homozygous mice walked with short pushes of the hindlimbs and performed rotarod and beam walking assays. In contrast to CMT2A patients, these hindlimb defects did not worsen with age.

Considering that CMT2A patients usually show wasting of the distal lower limb muscles of both the anterior and posterior compartments of the legs [34], Detmer and his colleagues investigated this symptom in the model. Homozygous animals showed a significant hindlimb muscle mass reduction (especially in the anterior region of the distal hindlimb) compared with wild-type and heterozygous animals; the posterior hindlimb muscle mass was comparable across all genotypes. Microscopic analysis of L4 and L5 motor roots demonstrated that homozygous transgenic animals had 40% fewer motor axons than wild-type animals. The motor axon loss was more severe in the small caliber axons. In this model, the forelimbs were never affected, in contrast to in CMT2A patients, who display upper limb involvement in 50% of cases [3]. Regarding sensory symptoms, homozygous and heterozygous *Mfn2*^T105M^ transgenic mice did not develop any sensory defects, even at 1 year of age. Ultrastructural studies of mitochondria in the peripheral nerves of CMT2A patients described abnormal mitochondrial aggregation in the distal part of axons [11, 20, 35] and corresponding impairment of the mitochondrial network and organization in the presence of *MFN2* mutations [21, 36]. These aggregated mitochondria with an improper distribution along the axons were also observed in transgenic homozygous mice. This improper mitochondrial recruitment could probably cause axonopathy.

Overall, these data confirmed the importance of this first mammalian model to reproduce several but not all clinical features of the disease, even if the weak points are not lacking, such as the presence of pathological phenotype only in a homozygous condition (in contrast with the dominant model of inheritance of the human disease) and the MN-restricted mutated MFN2 expression (while in patients, it is expressed everywhere).

### Eno *MFN2*^R94Q^ Transgenic Model

In 2010, heterozygous (MitoCharc1) and homozygous (MitoCharc2) transgenic mice with the R94Q amino acid substitution in human *MFN2* were generated by Cartoni and his group as a new CMT2A transgenic mouse model [37]. The mutated *MFN2* transgene was expressed under the control of a neuron-specific enolase (ENO) promoter, ensuring panneural expression both in the CNS and PNS [38–40]. The R94Q mutation, located in a hotspot region upstream of the MFN2 GTPase domain [7], appears to be the most common mutation in CMT2A patients with a severe phenotype [15, 41].

As a control, Eno *MFN2*^WT^ characterized by wild-type *MFN2* (*MFN2*^WT^) expression under the same promotor has been generated. Locomotor impairments and gait defects were evident in both 5-month-old mutant lines compared with *MFN2*^WT^ as demonstrated by the Rotarod test (MitoCharc1 versus *MFN2*^WT^, *P* < 0.001; and MitoCharc2 versus *MFN2*^WT^, *P* = 0.001) and the hind print test. In particular, motor phenotype of Mitocharc1 animals was more homogeneous than the MitoCharc2 mice’ one, likely for a non-complete penetrance effect. The MitoCharc2 mouse displayed also more severe phenotypical features than the MitoCharc1 mouse, such as a low body position, everted paws, and a dragging tail, aspects that could reproduce CMT2A patients’ difficulty in walking and tendency to fall [20]. Recently, Bernard-Marissal and his colleagues expanded the characterization of this mouse model, focusing only on Mitocharc1 mice. Confirming locomotor dysfunction observed by Rotarod, in-depth gait analysis showed also a significantly walking difficulty, with symptoms slightly progressing between 6 and 12 months. In particular, Mitocharc1 mice showed changes in the pressure and the surface of contact of the paw and in gait/posture and coordination monitored by CatWalk test. Despite locomotor impairment, no muscle strength alteration was observed at either 6 or 12 months by the grid test. However, the loss of neuromuscular junctions at the late stage of the disease was described without motoneuronal death. Regarding sensory characterization, the model did not show significantly sensory function impairment [25].

Concerning other remarkable pathological human features, such as alterations in mitochondrial morphology and distribution [20, 42, 43], 5-month-old MitoCharc1 mutant mice displayed an increase in mitochondria number (approximately 30%) in the distal part of sciatic nerve axons with diameters smaller than 3.5 μm, which were also overrepresented compared with those in controls [37]. Mitochondria alterations have been also observed by Bernard-Marissal and his group [25]. Live imaging of mitochondria in sciatic nerves of 1-month-old Mitocharc1 mice showed a reduction in mitochondria length as well as overabundance of small mitochondria due to an increase in the number of stationary mitochondria. These changes in morphology and motility could be due to reduced interactions between mitochondria and the endoplasmic reticulum (ER) at the level of mitochondria-associated membranes (MAM) as observed also in motorneurons’ soma in lumbar spinal cord of 12-month-old Mitocharc1 [25].

Electrophysiological analysis of MitoCharc1 showed a significant increase in the area/amplitude ratio for Aδ fibers during compound action potential recording in the sciatic nerves, confirming the observed functional axon alterations. This alteration seems to correlate with the increased proportion of small–medium caliber axons since the compound action potential area depends on the number of activated fibers and the square of their diameters [44].

Overall, Cartoni’s murine model represents a step forward from the HB9 *Mfn2*^T105M^ transgenic model [31], extending *MFN2* transgene expression to the nervous system and recapitulating some typical disease symptoms, such as locomotor impairment and axonopathy correlated to mitochondrial content alteration and transport. Mild-late pathological phenotype onset represents the main limitation of this model in contrast to severe-early clinical phenotype typically associated to CMT2A patients with *R94Q* MFN2 mutation.

### Nestin-cre *MFN2*^T105M^ Transgenic Model

In 2016, Bannerman and colleagues generated two other CMT2A mouse models, hemizygous for the expression of the human T105M missense mutation in the *MFN2* gene [45].

Unlike previous models [31, 37], to reproduce human pathological conditions, Bannerman extended mutated *MFN2* transgene expression to all cell types using a knock-in approach (Rosa-STOP-*MFN2*^T105M^) [46, 47]. Homozygous mice displayed wider clinical involvement characterized by multiple organ impairment, severe loss of motor function, and abnormal mitochondrial accumulation in Schwann cells in the sciatic nerve, compromising their survival.

To overcome this problem, Bannerman and his group decided to express the *MFN2* transgene only in neural and some non-neural cells, such as myosatellite cells, using the nestin-cre strategy [48, 49]. Since a clear length-dependent motor neuropathy is a clinical feature of CMT2A patients, the possibility of investigating how muscle and nerve interact in the presence of mutant MFN2 is very interesting for translational approaches.

Behavioral analysis showed no long-term difference between hemizygous nestin-cre MFN2^T105M^ mutant and control nestin-cre mice, as demonstrated by the Rotarod test, indicating apparently normal endurance, balance, and motor coordination. In contrast, an abnormality in the gait of these mice was observed through the Noldus Catwalk systems, and a statistically significant decrease in the print length of *MFN2* mutant mice was reported (control = 0.71 ± 0.072 cm, mutant = 0.58 ± 0.038 cm; *p* < 0.0003). These data could be linked to one of the main typical clinical features of CMT2A patients, the *pes cavus* [4, 50].

Despite a diminished number of mitochondria in peripheral nerve axons measured in tibialis muscle, these transgenic mice did not show aggregation or clumps of mitochondria in neuronal perikarya of MNs, even if no direct measures of axonal transport or alteration in mitochondrial distribution were performed. These findings contrasted with common MFN2 pathogenic features reported in other in vitro and in vivo disease models [10, 21, 23, 27]. Moreover, axon numbers, sizes, and g-ratios were not altered by *MFN2* transgene expression. Muscle fiber diameter was significantly altered in the soleus, involved in foot plantar flexion, and the tibialis anterior, involved in dorsiflexion actions, accompanied by decreased sarcomeric actin expression, disruption of striatal mitochondrial organization, and increased involvement of satellite cells in maintaining soleus muscle fiber composition.

Overall, the nestin-cre *MFN2*^T105M^ mouse model characterized by mutant MFN2 expression in a subset of cell types outside the PNS and CNS recapitulated some crucial phenotypical aspects of CMT2A patients (i.e., perturbations in foot gaiting and mitochondrial number reduction in tibialis muscle), but other aspects seem to be in contrast with other well-described studies. Furthermore, two aspects of this model limit its use for testing any therapeutic strategies: a mild late-onset motor phenotype and the absence of peripheral sensory loss.

### Thy1.2 *MFN2*^R94Q^ Transgenic Model

Recently, Zhou and colleagues generated the last transgenic mouse model of CMT2A, in which human *MFN2*^R94Q^ transgene was inserted in the Y chromosome and its expression was under the mouse Thy1.2 promoter [51]. As a control, Thy1.2 *MFN2*^WT^ characterized by wild-type *MFN2* (*MFN2*^WT^) expression under the same promoter has been generated. While the Eno promoter in the Cartoni mouse model [37] ensures expression principally in mature neurons, causing a mild late-onset phenotype (motor deficits start at 5 months of age), the Thy1.2 promoter should allow early transgene expression during the first postnatal week [52, 53], especially in projection neurons of the brain and spinal cord (mostly affected in CMT2A patients [54]). Previous works used this promoter for sensory nerve function studies and to visualize spine plasticity in the cortex and remodeling in peripheral synapses [55–57].

Thy1.2 *MFN2*^R94Q^ transgenic mice present stunted growth already visible at 2 months compared with Thy1.2 *MFN2*^WT^ and non-transgenic mice, while the survival curve showed that 25% of Thy1.2 *MFN2*^R94Q^ mice died by 15 months of age. Behavioral analyses indicated significant worsening of motor performance, with a minor latency to fall (Rotarod test) and weaker grip strength than *MFN2* ^WT^ or non-transgenic control mice.

To assess neurological dysfunctions such as vision loss and optic atrophy sometimes observed in CMT2A patients [2, 58], the measurement of visual acuity by the optokinetic response test (OKR) and optical nerve analysis was performed in this mouse model. Thy1.2 *MFN2*^R94Q^ mice showed pronounced vision loss associated with significant neurofilament loss and axonal spheroids.

Considering that another important CMT2A human phenotype feature includes spinal cord involvement with spastic paraparesis and brain white matter changes [5, 59, 60], neuronal and axonal damage and degeneration have been investigated in Thy1.2 *MFN2*^R94Q^ mice in both the central and peripheral nervous systems. Five-month-old animals showed axon atrophy and deterioration without neuronal cell body loss, especially in pyramidal tracts of the medulla and lumbar spinal cord, but not in the distal tibial nerve, as already observed in previous studies [15, 61, 62].

Regarding the abnormalities in mitochondrial morphology, size, and distribution identified in CMT2A patient nerve biopsies [20, 42], Thy1.2 *MFN2*^R94Q^ mice showed fragmented mitochondrial clumps in neuronal cell bodies and proximal and distal axons, which were positive for the mitophagy markers p62 and ubiquitin. Similar data have already been described in iPSC-derived MNs from CMT2A patients [10] and in other culture models [18, 24] but never in an animal disease model.

Overall, this last mouse model reproduced reliable pathological features such as severe early-onset sensorimotor deficits, vision loss, and widespread axonal degeneration with altered mitochondrial dynamics. The insertion of the *MFN2* transgene into the Y chromosome limited the analysis only to male mice, although to date, clinical data from CMT2A patients have not highlighted sex differences in phenotypes.

### A Rat Model of CMT2A: a Valid Alternative to Mouse Models?

Although mice have usually been the primary model of choice for most research, recent advances have expanded the testing capabilities of the rat, whose larger size and varied characteristics allow for greater procedural manipulation better suited for many newly emerging therapeutic areas.

Recently, a rat model of CMT2A carrying the R364W mutation in the *Mfn2* gene has been generated (http://www.psychogenics.com/abstract67.html). This model is characterized by multiple motor deficits that worsen over time. Regarding caudal nerve conduction, mutant rats showed a progressive decline in the amplitude of the compound action potential after 20 weeks. Finally, the authors observed a reduced density of myelinated axons and active axonal degeneration in distal but not proximal nerves. Overall, a genetically authentic animal model of CMT2A that develops a progressive, length-dependent axonal neuropathy could be a valuable tool for examining the pathogenesis and treatment of CMT2A.

### Therapeutic Implications

The importance of developing accurate disease models that recapitulate the molecular and clinical features of human disease stands out while attempting to evaluate novel therapeutic strategies. Indeed, these characteristics are necessary for researchers to assess the real treatment efficacy in vivo and to justify research translation from the bench to the bedside. An inaccurate animal model might lead to the impossibility of validating outcomes due to insufficient phenotypical expression or to marked discrepancy between preclinical and clinical results.

Considering CMT-type diseases, no effective therapy has reached the clinic so far, although some strategies have obtained promising results in animal models. Rocha and colleagues tested mitofusin agonists (MAs), which are small molecules that mimic the MFN2 peptide–peptide interface and are able to allosterically activate the protein, thus promoting mitochondrial fusion [63]. MAs were administered to cultured neurons expressing *MFN2*^R94Q^ and *MFN2*^T105M^ and to *MFN2*^T105M^ transgenic mice via sciatic nerve injection, proving able to improve mitochondrial dysmotility, fragmentation, and clumping in vitro and restoring mitochondrial motility to normal levels in vivo*.*

Encouraging results also come from studies regarding inhibitors of histone deacetylases (HDACs), a family of enzymes that catalyzes the deacetylation of conserved serine residues in histones and non-histone proteins. HDAC6 is a member of this class that acts mainly at a cytoplasmic level, and it was shown to be responsible for the deacetylation of alpha-tubulin, a component of the cytoskeletal network. Since an increase in HDAC6 activity seems to be implicated in the pathogenesis of CMT2F [64], caused by mutations in *HSPB1*, d’Ydewalle and colleagues first tried HDAC6 inhibitors in a CMT2F transgenic mouse model, and the inhibitors corrected the axonal transport defects caused by *HSPB1* mutation and reversed the CMT phenotype [65]. In addition, HDAC6 inhibition also appears to be capable of improving disrupted mitochondrial fusion and mitochondrial transport [66, 67], and from this observation stemmed the idea of testing this strategy in CMT2A models as well. Indeed, in a recently published report, Picci and colleagues investigated the effect of pharmacological HDAC6 inhibition on MitoCharc1 mice [68], showing that the treatment was able to prevent alpha-tubulin hypoacetylation and rescue motor performance at both the pre-symptomatic and post-symptomatic stages.

These results carry relevant implications, as they shed light on interesting pathogenic mechanisms, revealing unexpected connections between genetically different neuropathies and between mitochondrial dysregulation and cytoskeletal impairment. Furthermore, they pave the way for future therapeutic trials in CMT2A patients.

## Conclusion

**Fig. 1:**
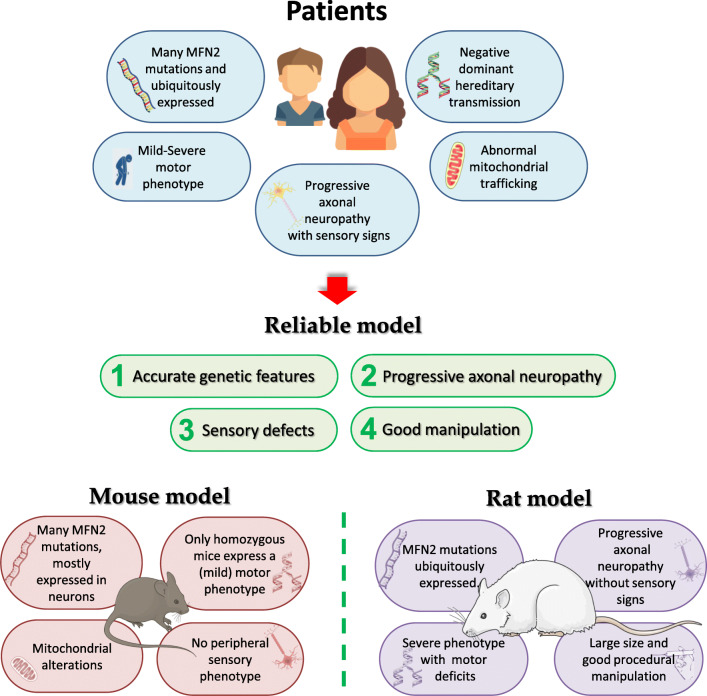
Reliable CMT2A disease model: integrating data from disease animal models and patients

CMT2A is an inherited, debilitating, sensory motor neuropathy caused by missense mutations in the *MFN2* gene with an autosomal dominant pattern of inheritance [7, 15–19]. Currently, no cure is available for this devastating disorder. Nevertheless, few studies on the development of potentially effective therapies are currently ongoing. One of the major obstacles in developing effective therapy, as well as in investigating disease pathogenesis, is the lack of reliable animal models that show accurate genetic features and develop progressive, length-dependent axonal neuropathy (Fig. 1).

Based on our literature review of CMT2A animal models, there are still many aspects to be investigated. The expression level and pattern of expression of the causative *MFN2* gene represent the principal technical limitations of available mouse models. As pointed out from Table 1, these models are transgenic models overexpressing mutant MFN2, usually via a human transgene. In some models, the expression is rather restricted to the nervous system by specific promoters, while in others, transgene expression is ubiquitous. The question remains as to whether a physiological level of the mutant MFN2 is necessary to sufficiently comprehend the pathogenetic mechanism. Will humanized mice with a human *MFN2* transgene randomly integrated into the mouse genome have the same regulatory mechanism as patients? Can overexpression models adequately mimic the disease pathology?

Among models with a CMT2A autosomal dominant pattern, motor deficits were only observed in the homozygous mouse model. The heterozygous mice with an allelic pattern similar to the patient failed to develop a disease phenotype, which might be due to the differences between mice and humans in terms of life span, disease duration, and exposure to different environments. Another surprising aspect was that heterozygous mice displayed no disease progression or increasing severity on aging. Does such a finding suggest that the short lifespan of mice is insufficient to reproduce late-onset neuropathy? To date, it is not known why some mice, although carrying a pathogenic mutation expressing the mutation, do not show the pathological phenotype. It is also possible that these so-called pathogenic mutations may act synergistically with other unknown genetic and epigenetic modifiers that could potentially accelerate or delay the phenotype.

The complexities of CMT2A are clearly exemplified by the wide array of human clinical phenotypes and by the lack of effective phenotype–genotype correlation [35]. Considering that more than 100 different MFN2 mutations have been reported and that mutations in the same codon often result in different amino acid substitutions [69] correlating to clinical phenotypes with different severities, one open question is whether it is possible to develop an in vivo model that would collectively reflect all the features described in patients with various mutations. On the other hand, the generation of a preclinical model for each identified mutation is not feasible but remains crucial to find an effective therapy. The creation of a unique mouse model overexpressing a single *MFN2* mutation associated to a severe phenotype with early onset could allow to model the most severe hallmarks and find a therapy that can likely be suitable for the milder phenotypes. Moreover, if the mouse model obtained by the overexpressing of a single severe mutation exhibits common motor and sensory phenotypes observed clinically in CMT2A, it can be considered as a proper model also for the other mutations as occurs in other neurodegenerative diseases (i.e., SOD1G93A ALS mice). The research into CMT2A, which has been performed relatively recently compared with research into other neurodegenerative diseases, has taught us that to improve our understanding of disease pathogenesis, the community must embrace the disease complexities and work with different models (Fig. 1). Integrating data from multiple sources, mice and humans, in vivo and in vitro, should allow us to build a reliable disease model.
